# Wheat Breeding through Genetic and Physical Mapping 2

**DOI:** 10.3390/ijms222413359

**Published:** 2021-12-12

**Authors:** Agata Gadaleta

**Affiliations:** Department of Agricultural and Environmental Science, University of Bari Aldo Moro, 70126 Bari, Italy; agata.gadaleta@uniba.it

Following the success of the first topic, the special issue of “Wheat breeding through genetic and physical mapping 2” has been re-proposed in order to keep current the recent advancement in research on genetic and physical mapping of candidate genes for agronomically important traits, in studies of the regulatory sequence for biotic and abiotic stress resistance. The special issue includes a total of nine published papers, of which seven were original research manuscripts and two were review papers. 

Tong et al. [1] summarized molecular processes and genes involved in Fe and Zn homeostasis in the model plants arabidopsis and rice, identified their orthologs in the wheat genome, and related them to the known wheat Fe/Zn quantitative trait locus/loci (QTL), based on physical positions. The current study provided the first inventory of the genes regulating grain Fe and Zn homeostasis in wheat, which will benefit gene discovery and breeding and thereby may accelerate the release of Fe- and Zn-enriched wheat breeding lines. 

Biofortification is an important tool in wheat. Several research studies have been conducted in order to increase fiber content [2]; the results of glucan breeding in wheat were summarized in the review of the first edition of the special issue by Marcotuli et al. [3]. The review by Ashraf et al. [4] reported an overview of the possibility of using male sterility (MS) systems as an important platform for improvement in agriculture production. MS systems have been used to create bulk germplasm of the two-line hybrids (EGMS) in rice, to gain production sustainability and to exploit their immense potential. This article provided a deep understanding of the molecular control of MS in EGMS lines and explored the regulatory driving forces that function efficiently during plant adaption in a changing environment. The authors highlighted a possible solution in obtaining more stable hybrids through apomixis (a single-line system) for seed production.

The seven remaining research articles were focused on yield and related traits. A study showing the detection of yield-related QTL clusters and the potential candidate genes in two wheat DH populations were reported by Zhang et al. [5]. This study may provide new insights into specific genome regions for major gene predictions. The construction of fine consensus maps was obtained using 12,000 GBS SNP markers. Twenty-two traits were evaluated, and 227 QTL were detected after combining several traits, different environments, and neighboring individual QTL. In addition, potential candidate genes in each QTL cluster were predicted and validated. The QTL clusters with positive linkages between traits or without negative impact were suggested for use in wheat breeding. 

On the same topic, Ren et al. [6] studied a candidate gene for the yield component of grain weight and size in bread wheat. The authors identified five splicing variants of the Gγ subunit gene TaGS3 (TaGS3.1 to TaGS3.5), which showed expression divergence during wheat polyploidization and a differential function in grain weight and size determination. TaGS3.1 overexpression significantly reduced grain weight by 5.89% and grain length by 5.04%, while TaGS3.2–3.4 overexpression did not significantly alter grain size, when compared to wild types. The results indicate that TaGS3 differentially regulates grain size via AS, by which the regulation of grain size is fine-tuned and balanced at the post-transcriptional level.

Yield is a very important trait, strongly depending on environmental conditions, and the characterization of wild genetic materials is a key topic for plant breeding. Chidzanga et al. [7] indicated that exotic germplasm is a rich source of genetic diversity; however, it harbors undesirable traits that limit its suitability for modern agriculture. Nested association mapping (NAM) populations are valuable genetic resources that enable incorporation of genetic diversity, dissection of complex traits, and the provision of germplasm in breeding programs. The authors reported data on the development of the OzNAM population by crossing and backcrossing 73 diverse exotic parents to two Australian elite varieties, Gladius and Scout. In total, 3535 BC1F4:6 RILs from 125 families, with 21 to 76 lines per family, were genotyped, and the authors found 4964 polymorphic and multi-allelic haplotype markers that spanned the whole genome. A subset of 530 lines from 28 families were evaluated in multi-environment trials over three years. The multi-parent property of NAM populations allows for the detection of QTL with multiple alleles. QTL for maturity and plant height were identified. 

Yacoubi et al. [8] reported work on the ARS gene family, which was found to be involved in abiotic stresses. They characterized two ABA-WDS domains, isolated from durum wheat (TtABA-WDS) and barley (HvABA-WDS). Bioinformatics analysis showed that they are both consistently predicted to be intrinsically disordered. Hydrodynamic and circular dichroism analysis indicated that both domains are largely disordered but belong to different structural classes, with HvABA-WDS and TtABA-WDS adopting a PreMolten Globule-like (PMG-like) and a Random Coil-like (RC-like) conformation, respectively. In the presence of the secondary structure stabilizer trifluoroethanol (TFE) or of increasing glycerol concentrations, which mimic dehydration, the two domains acquire an α-helical structure. Interestingly, both domains are able to prevent heat- and dehydration-induced inactivation of the enzyme lactate dehydrogenase (LDH). The results presented would support a reconsideration of the ABA-WDS family as a member of the LEA superfamily. The same candidate-gene approach was reported by Nigro et al. [9] as validating the involvement of glutamine synthetase and glutamate synthase genes in the control of high GPC in durum wheat. A useful and efficient method of validating a putative QTL is the constitution of near-isogenic lines (NILs), by using the marker found to be associated with QTL. In this special issue, the authors presented the development of two distinct sets of heterogeneous inbred family (HIF)- based NILs segregated for GS2 and Fd-GOGAT genes obtained from heterozygous lines at those loci, as well as their genotypic and phenotypic characterizations. The results allowed the validation of the previously identified GPC QTL on 2A and 2B chromosomes, together with the role of these key genes in GPC control.

The next two papers studied the wheat-wall-associated kinase and like kinase receptors involved in pathogen resistance. Guo et al. [10] wrote about the roles of the wheat-wall-associated kinases (WAKs) in defense against both *F. graminearum* and *R. cerealis*, which have remained largely unknown. This research reported the identification of TaWAK2A-800, a wheat WAK-coding gene located on chromosome 2A, and its functional roles in wheat resistance responses to FHB and sharp eyespot. The TaWAK2A-800 transcript abundance was elevated by the early infection of *R. cerealis* and *F. graminearum*, or treatment with exogenous chitin. The gene transcript seemed to correspond to the resistance of wheat. One of the first reports on the involvement of WAK genes against fusarium in wheat was from Gadaleta et al. [11]. They carried out a map-based cloning of QFhb.mgb-2A, identifying a WAK2 gene responsible for fusarium head blight resistance in wheat; the involvement of the WAK2 gene in the FHB resistance mechanism was assessed by a gene expression comparison between resistant and susceptible wheat lines, and by a disease-symptom evaluation in 3 TILLING mutants for WAK protein function. In the study by Qi et al. [12] of a WAK gene TaWAK7D, located on chromosome 7D, the authors showed its positive regulatory role in the defense response to *R. cerealis* infection in wheat. RNA-seq and qRT-PCR analyses showed that TaWAK7D transcript abundance was elevated in wheat after *R. cerealis* inoculation, and the induction in the stem was the highest among the tested organs. Additionally, TaWAK7D transcript levels were significantly elevated by pectin and chitin treatments. The knock-down of TaWAK7D transcript levels impaired resistance to *R. cerealis* and repressed the expression of five pathogenesis-related genes in wheat. The studies of these gene families, reported in the above-cited articles, may provide an important contribution to studies of wheat pathogen resistance.

The present special issue was very successful and was concentrated on the use and identification of important candidate genes for several traits, such as yield, grain protein content, and pathogen resistance. We wish to thank all the authors for their significant contributions to this article’s collection, and to thank the International Journal of Molecular Science for its support. 

## Data Availability

Not applicable.
